# Real-time Particle Size Analysis Using the Focused Beam Reflectance Measurement Probe for *In Situ* Fabrication of Polyacrylamide–Filler Composite Materials

**DOI:** 10.1038/s41598-019-46451-x

**Published:** 2019-07-12

**Authors:** Sivashunmugam Sankaranarayanan, Blaž Likozar, Rodrigo Navia

**Affiliations:** 1 Scientific and Technological Bioresource Nucleus (BIOREN), Universidad de La Frontera, Av. Francisco Salazar 01145, Temuco, 4780000 Chile; 2 Department of Catalysis and Chemical Reaction Engineering, National Institute of Chemistry, Hajdrihova 19, SI-1001, Ljubljana, Slovenia; 3 Department of Chemical Engineering, Faculty of Engineering and Sciences, Universidad de La Frontera, Av. Francisco Salazar 01145, Temuco, 4780000 Chile; 4 Centre for Biotechnology and Bioengineering (CeBiB), Universidad de La Frontera, Av. Francisco Salazar 01145, Temuco, 4780000 Chile

**Keywords:** Materials science, Composites

## Abstract

Real-time particle size analysis, using an engineered focused beam reflectance measurement (FBRM), was studied for the fabrication of chemical composite materials, applying various (inorganic/organic/biological) filler powders with polyacrylamide *via* the *in situ* polymerization production process at 80 °C for 24 h. The measured diameter dimensions, differential distribution functions and growth during reactive compound manufacturing technology were monitored by determining quantitative chord length, this being the altering scale use of FBRM technique. Materials characterizations such as formulation part-, scanning electron microscopy-, substance elemental- and complex Fourier-transform infrared spectroscopy analyses, supported well the successful structural preparation of differing-property constituent compositions. In addition, it was also observed that operations such as granulation, coating and filling, were involved in the design of stronger polymer–reinforcement components. A comparison of the surface area variation of montmorillonite (245 m^2^/g), alumina (236 m^2^/g) and residual biomass (0.8 m^2^/g) with their corresponding formed composites (112, 84 and 0.1 m^2^/g, respectively) revealed that the presence of thermoset plastic matrix results in a drop in interface due to a defined multiple step formation processing. Furthermore, thermal characterization of alumina and the developed nanocomposite materials confirmed, as expected, the interaction of the nanocomposite precursors.

## Introduction

Composite materials are nothing but the combination of two or more components in which the physical and chemical properties of the developed composite materials are defined by its individual components^1^. Methods such as sol-gel process^2^, building block approach^3,4^, co-solution method^5^, *in situ* processing of components^6,7^, post-synthetic modifications^8^ and templated synthesis^9^ are some of the possible approaches for the development of composite materials. In recent years, preparation of inorganic**–**organic composite materials by combining inorganic particles along with organic polymers gained more interest in the field of advanced functional materials^10^. Here, different types of inorganic filler particles (e.g. nanoparticles, nanofibers, fragments) can be incorporated with the polymer components which acts as a matrix and results in polymer–filler composite materials with improved mechanical strength, electrical conductivity, thermal resistance^11–13^. In the structure of these type of polymer–filler composites, fillers surround and bind together with the material (polymers) matrix^14^. Their properties depends on various parameters such as nanostructure design, processing methods and sintering techniques whereas the desired product formation can be affected by hydrophilicity, surface roughness, and contact angle of the components^15^. In conventional processes, fusion of carbon nanoparticles (as filler) in polymers may result in plastic deformation due to the reduction in polymer resistance by permitting cavitation and bond breaking activities^16^. This clearly indicates that particles content plays a vital role than adhesion behaviour for the alteration in the composite stiffness. Recent report on electrospun process towards the preparation of nanofiber based composites (PVDF nanofibers with thermoset composite laminates) revealed that during laminates curing process, PVDF material also undergoes phase transformation which affects the performance of the composites^17^. To overcome these drawbacks, novel advanced processes are highly in demand at this stage which can also give clear understanding of composite formation mechanisms. Apart from conventional heating, some of the advanced novel approaches such as electron beam, gamma or X-rays, ultraviolet (UV) and infra-red (IR) radiations, microwave and radiofrequency heating are used for accelerated curing of thermoset polymers and composites^18^.

Due to their flexible properties, polymer–inorganic composite materials finds various applications including medicine, food packaging, cosmetics, textiles, agriculture, optoelectronics, automotive industries, membranes, microelectronics, biocompatible and aerospace materials, batteries, electrochemical display devices and electrical-magnetic shields^15,19–21^. In general, polymer–inorganic composite materials synthesis may follow different synthetic approaches such as granulation^22^, coating^23^, encapsulation^24^ and filling process^25^ and all these methods has the advantages such as simple preparation, low cost and possibility for easy scalable process. Adherence of solids or liquids on primary powder materials is called granulation or beads formation in which particle size, density and flow process can be managed to obtain granules of multiple components. Coating is the process where the surface of the filler particles is covered by polymer components and mostly this process will happen in a two-dimensional approach. Encapsulation follows the concept of improving the stability as well as life time of a molecule being protected by a one component matrix. In general due the difference in surface nature and poor interfacial adhesion, inorganic filler particles have low compatibility with polymer matrix^26,27^. In order to improve the interaction between organic and inorganic components, adhesive promoters with amphiphilic molecules were reported^28^. So filler particles with variations in sizes as well as surface properties can have a huge influence on the reactivity/adherence with the polymer matrix and that may alters the formation of the composite materials. Preparation of different polymer–filler composite materials by carry out *in situ* polymerization is an interesting approach because this process may follow entirely different mechanism in the formation of composite materials. Acrylamide is a well-known water soluble monomer source for the synthesis of polyacrylamide by free-radical polymerization in presence of an initiator and cross linker. Acrylamide has favourable reactivity with many co-monomers and polyacrylamide is able to be derivatized with various polymers with a wide range of molecular mass, charge densities, and chemical functionalities. In addition, large amount of heat develops during the polymerization process which can result in rapid temperature rise^29^.

**Figure 1:**
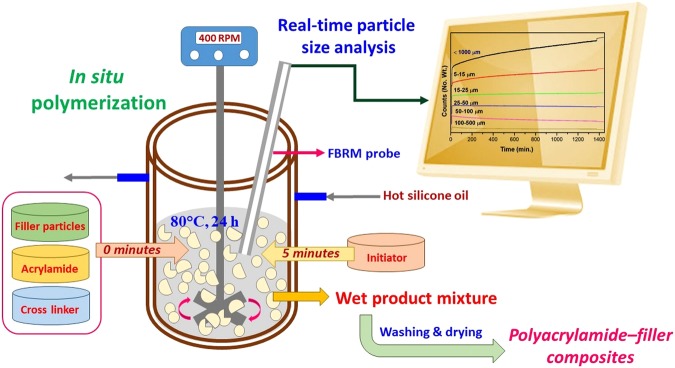
Pictorial representation of real-time particle size analysis using FBRM technique for the fabrication of polyacrylamide–filler composites by *in situ* polymerization process.

Focused beam reflectance measurement (FBRM) is a real-time (on-line) monitoring tool for the determination of size and shape of the particles in the process by considering the chord length of the formed particles. Using this technique, some studies have been already reported for monitoring and controlling the crystallization process^30^, solidification of micro-particles^31^, granulation^32^ and granulation-drying-milling process^33^. FBRM technique has a straight relationship with chord length distribution which is influenced by the geometry, size, number and dispersion of the particles. So, FBRM technique can be effectively used for the determination of the particle size changes kinetics in the fabrication of composite materials, giving the best understanding about the material formation mechanism. The aim of this work was to study the particle size changes kinetics of commercially available filler particles in the *in situ* preparation of polymer–filler composite materials using on-line FBRM techniques. Pictorial representation of real-time particle size analysis using FBRM technique for the fabrication of polyacrylamide–filler composites by *in situ* polymerization process is shown in Fig. 1. For the *in situ* polymerization process, acrylamide was chosen as monomer source to get the water soluble polyacrylamide as matrix in the system. As a consequence, the formed polyacrylamide in aqueous solution blended with different filler particles for the fabrication process of polyacrylamide–filler composites. To the best of our knowledge, this is the first report on the real-time particle size analysis using FBRM technique for the synthesis of various polyacrylamide–filler (inorganic/organic/biological) composite materials by *in situ* polymerization process using acrylamide as monomer source.

## Results and Discussion

### Real-time particle size and particle size distribution analyses

**Figure 2:**
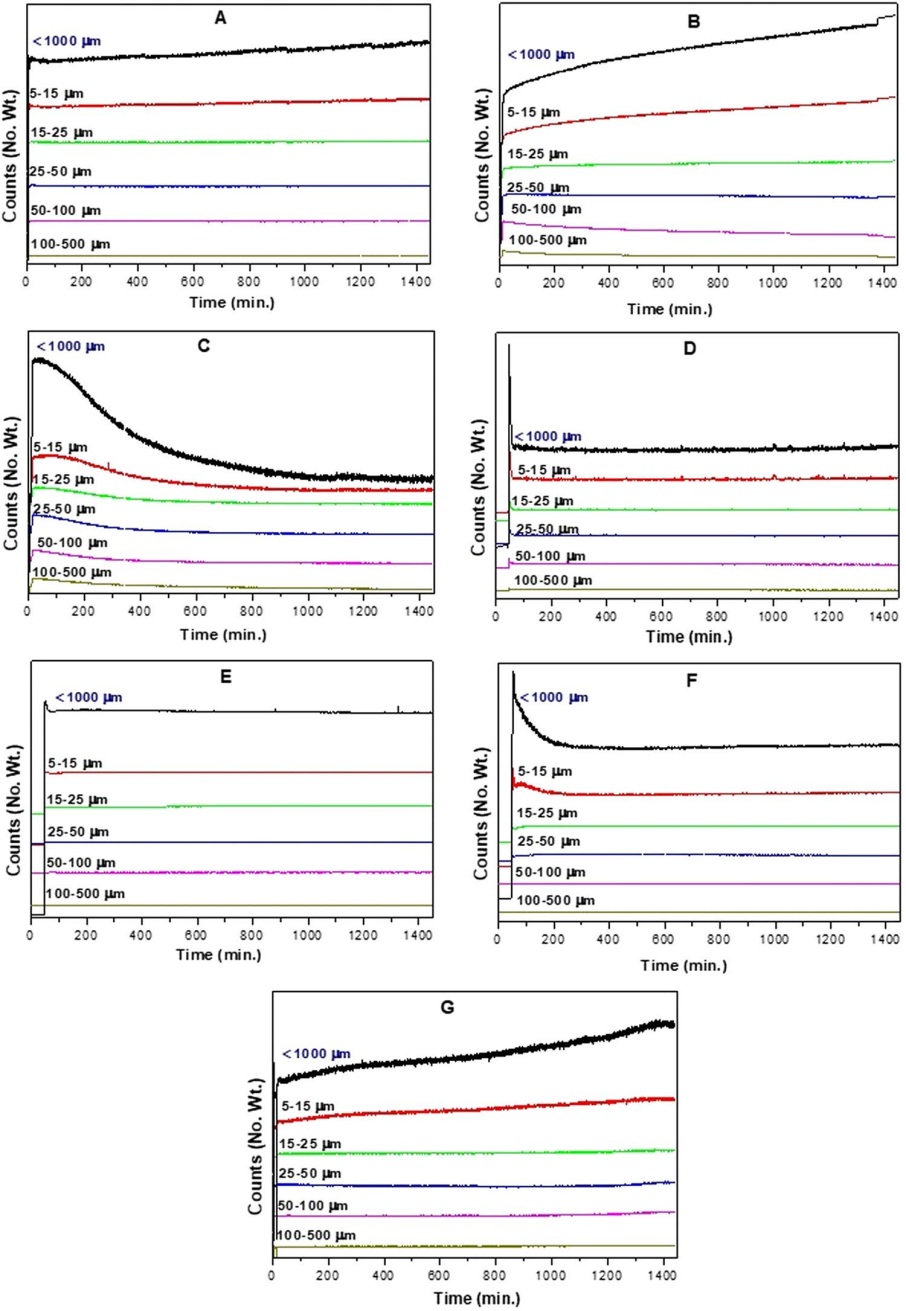
Real-time particle size analysis of polyacrylamide–filler composites preparation via *in situ* polymerization using: (**A**) montmorillonite, (**B**) alumina, (**C**) silica, (**D**) zeolite Y, (**E**) titania, (**F**) activated carbon, (**G**) residual biomass as filler particles with acrylamide as polymer precursor.

**Figure 3:**
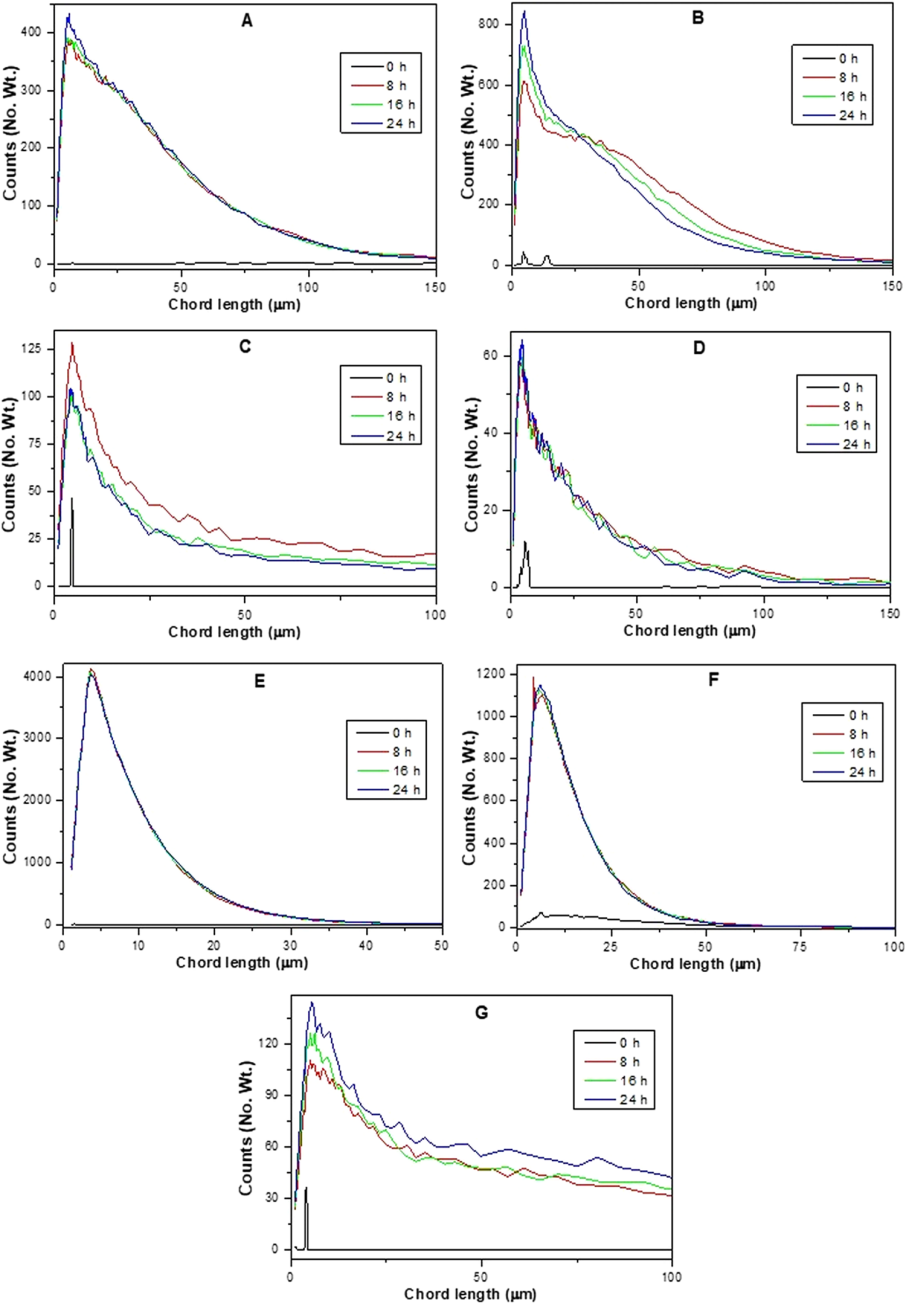
Real-time particle size distribution of polyacrylamide–filler composites preparation via *in situ* polymerization using: (**A**) montmorillonite, (**B**) alumina, (**C**) silica, (**D**) zeolite Y, (**E**) titania, (**F**) activated carbon, (**G**) residual biomass as filler particles with acrylamide as polymer precursor.

Commercial inorganic adsorbents such as alumina, montmorillonite, silica, zeolite Y and titania, as well as activated carbon (organic adsorbent), were used as filler particles for the preparation of polymer–filler composite materials by *in situ* polymerization process using acrylamide as a polymer precursor. As a initial step, polymerization reaction was optimized separately by taking acrylamide, N,N′-methylene bisacrylamide and ammonium persulfate in water medium. Then the same condition was adopted for the preparation of polyacrylamide–filler composite materials in presence of various filler particles. FBRM probe in water medium at 80 °C showed zero counts of particles without the interference of bubbles formation during high-speed stirring, suggesting the accuracy of the probe. The counts started to increase after the addition of the reactants to the system. In all the cases, real-time particle size analysis was performed using FBRM technique by measuring the chord length which counts from 15 to 1000 micrometers (μm). Real-time particle size analysis and particle size distribution for the preparation of various polymer–filler composite materials by *in situ* polymerization process from 0 to 24 h are shown in Figs. 2 and 3, respectively.

Composite materials prepared by using montmorillonite (A) and alumina (B) as filler particles with *in situ* generated polyacrylamide as organic representative showed increased counts for particles with a size of less than 1000 μm. In both the materials, changes were mainly observed only in the range between 5 and 15 μm whereas remaining particle size regions were almost constant during the whole process. These results suggests that compared to original raw materials which are in the range between 5 and 15 μm, the developed composites resulted in slightly larger sized particles during the polymerization process, most probably following a granulation mechanism. In these cases, the fillers may act the as core material while the *in situ* formed polyacrylamide may play a role as a support shell material. In the case of montmorillonite, most of the particles were in the range of 5–50 μm and the particle size distribution increased with the process time. Also, after 24 h, maximum particles count was in the size of 5–25 μm only. This shows a uniform particle size distribution and also homogeneity of the larger particles when using montmorillonite as filler particles. In the case of alumina, the particle size distribution pattern follows the same trend as montmorillonite. After 8 h, polyacrylamide–alumina composites showed moderate amount of particles in the range between 50 and 100 μm, but after that the particle size decreased probably due to stirring effects. Though the drop in particle size observed in the range between 50 and 100 μm, more uniformly sized particles of 5–15 μm were observed, indicating a breakdown followed by a granulation process.

In case of silica (C) as filler particles, the real-time particle size analysis followed a different pattern compared to the previously discussed cases. The decrease in the particle size below 1000 μm during the process may be a result of the dispersion of silica or the occurrence of precipitates/solidification in the system, making the particle size analysis by the FBRM probe more difficult. Particle size distribution indicates that after 16 h, further increase in the time doesn’t have influence in the particle size. Though the particle sizes (5–15 μm) are uniform at different times, decrease in the counts were observed with higher time. So, silica as filler particles may have followed a coating or filling process, which can certainly change the dispersion of the medium in the preparation of polymer based composite materials. Zeolite Y (D) as filler particles, showed a constant maintenance of particle size during the corresponding polymer based composite material preparation process. Though the results show no change in the overall particle size of less than 1000 μm, slight increase in the particle size was observed in the region of 5–15 μm which may obeyed to a granulation mechanism. To support this argument, the particle size distribution pattern of these materials looks similar to the previous observations in the case of montmorillonite and alumina as filler particles. The particle size reached an optimum at 8 h and then remained constant with further increments in the process time. These results indicate that the polymerization process happened in the region of 5–15 μm without any influence on the overall particle size distribution. Using titania (E) as a filler particles, particle size increased constantly in the region of 5–15 μm, supporting that the composite material formation process obeys a granulation mechanism. But a close observation in the region of less than 1000 μm showed a sequential increase and decrease in particle size, probably related to an agglomeration process, followed by the deformation of the materials. In general, deformation may not be possible in this case because no drop in particle size was observed in the 5–15 μm region. Particle size distribution studies revealed that after 8 h more uniformed particles in the region of 5–10 μm were formed and then remained same till the end of the process (24 h). So, the drop in particle size may be related to the lack of dispersion or precipitation/solidification of the formed products which may result difficulty in FBRM analysis, as observed before with silica particles.

In the case of activated carbon (F) as filler particles, the particle size analysis pattern follows a different trend than previous studied filler particles. Initially, the particle size decreased to reach the minimum and then increased very slowly until the end of the process time. The particle size distribution analysis showed a slight increase in the chord length from 8 to 24 h reaction time. All these changes were observed in the region of 5–25 μm which supports the homogeneity of the particle sizes in the samples. The initial drop in the particle size may be related to the fast dispersion of activated carbon in water medium which is completely different from inorganic filler particles used in this study. Once polymerization process started, granulation took place, resulting in a minor change of the particle size in the region of 5–15 μm. The study was further extended to the preparation of polyacrylamide–residual biomass (G) composite material. The particle size of the polyacrylamide–residual biomass composite material preparation process increased continuously with the increase of reaction time. Continuous increase in the particle size in 5–15 μm region shows that granulation/agglomeration of larger particles of residual biomass happened, with *in situ* formed polyacrylamide. It was observed that pores were created in the prepared polyacrylamide–residual biomass composite material due to the removal of some of the organic moieties (evidenced by colour change to brown) at the reaction temperature. Particle size distribution of polyacrylamide–residual biomass composite material follows the same trend as alumina. After 8 h, polyacrylamide–residual biomass composite material showed moderate amount of particle between 0–25 μm range and the same increased with the process time.

Real-time particle size analysis and particle size distribution studies of all of the filler particles with *in situ* polymerization clearly supported the formation polyacrylamide based composite materials. Interestingly, these results indicate that all the materials followed different composite formation mechanisms, depending on the size of the filler particles as well as polymer–filler interactions.

### Elemental Analysis

In order to further confirm the polyacrylamide–filler composite materials formation, elemental (CHNS-O) analyses were performed and the results are given in Table 1. In all the prepared composite materials, carbon, hydrogen and nitrogen content values increased compared to neat filler particles, indicating the change in the compositions due to polymerization process. The inorganic filler particles such as montmorillonite, alumina, silica, zeolite Y and titania did not show any C and N contents whereas their corresponding polymer composites showed the presence of C and N. Presence of N in all formed product composite materials confirms the successful formation of the composites. When using residual biomass with an N content of 1.45% related to proteins presence, the polymer–residual biomass composite materials resulted in an increased N content of 3.18%, supporting the argument of successful polymer–filler composites formation via *in situ* polymerization. CHNS-O analyses of all the composites supported FBRM observations on composite materials formation.

**Table 1:** Elemental analysis of the neat filler particles and the prepared polyacrylamide–filler composites.

Name of the material^a^	C (%)	H (%)	N (%)	O (%)	Total (%)
Montmorillonite	—	1.4	—	98.6	100
Polyacrylamide–montmorillonite composite	7.4	2.7	2.7	87.2	100
Alumina	—	3.8	—	96.2	100
Polyacrylamide–alumina composite	9.2	5.2	3.3	82.3	100
Silica	—	—	—	100	100
Polyacrylamide–silica composite	11.4	2.6	4.3	81.7	100
Zeolite Y	—	1.1	—	98.9	100
Polyacrylamide–zeolite Y composite	4.8	2.7	1.8	90.7	100
Titania	—	—	—	100	100
Polyacrylamide–titania composite	8.7	1.9	3.5	85.9	100
Activated carbon	75.4	—	—	24.6	100
Polyacrylamide–activated carbon composite	82.6	2.2	2.2	13.0	100
Residual biomass	27.5	5.3	1.5	65.7	100
Polyacrylamide–residual biomass composite	39.0	6.4	3.2	51.4	100

^a^Sulphur content was not observed in all the samples.

### Scanning electron microscopy studies

**Figure 4:**
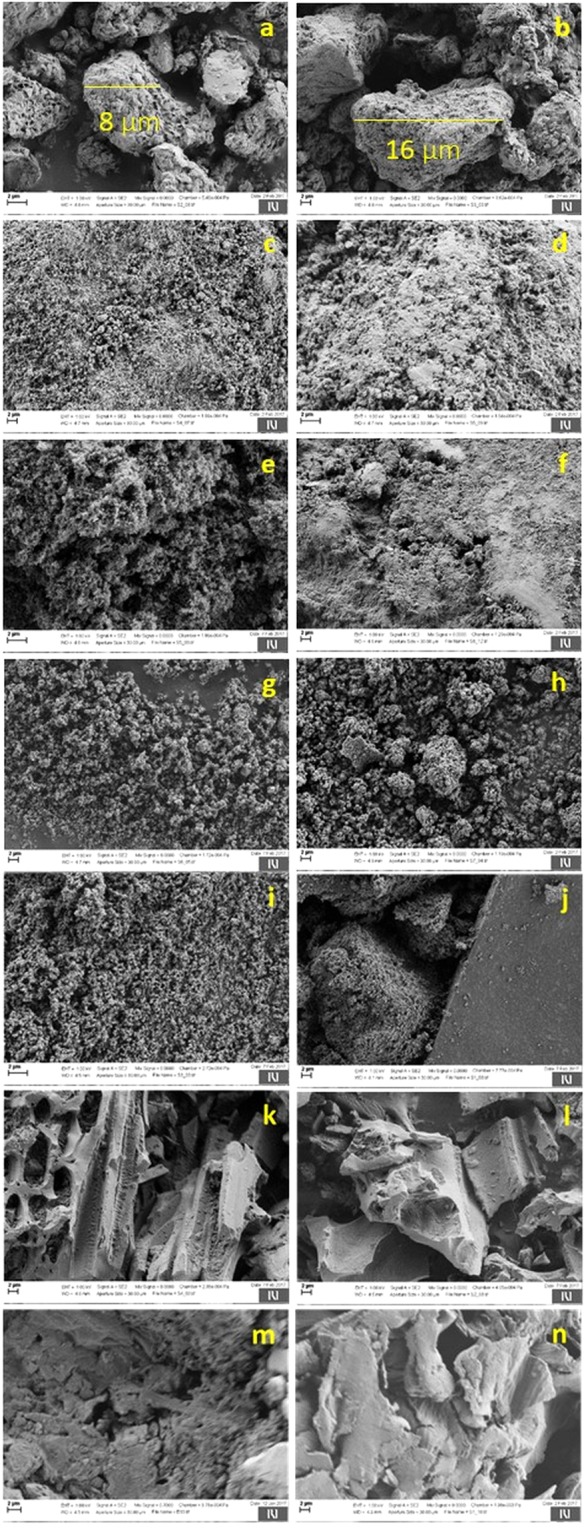
SEM images of (**a**) montmorillonite, (**b**) polyacrylamide–montmorillonite composite, (**c**) alumina, (**d**) polyacrylamide–alumina composite, (**e**) silica, (**f**) polyacrylamide–silica composite, (**g**) zeolite Y, (**h**) polyacrylamide–zeolite Y composite, (**i**) titania, (**j**) polyacrylamide–titania composite, (**k**) activated carbon, (**l**) polyacrylamide–activated carbon composite, (**m**) residual biomass, (**n**) polyacrylamide–residual biomass composite.

Scanning electron microscope (SEM) images of neat filler particles and the prepared polyacrylamide–filler composite materials are shown in Fig. 4. In the case polyacrylamide**–**montmorillonite composite materials (Fig. 4b), the resulted particles are larger in size (16 μm; nearly 2 times) than montmorillonite (8 μm; Fig. 4a) and also showed a completely different surface structure. These results indicate the coating of *in situ* generated polyacrylamide on the montmorillonite surface, supporting the successful formation of composite materials as concluded from FBRM and elemental analyses. The surface of alumina (Fig. 4c) showed a small bead type particles on it whereas polyacrylamide**–**alumina composite material (Fig. 4d) showed a completely different type of surface which is occupied by some foreign compounds. The polyacrylamide–alumina composite material surface might have undergone granulation on the one hand, and filling by polyacrylamide moiety on the other hand, resulting in a unique composite material. The surface of silica (Fig. 4e) showed a spongy like morphology by the agglomeration of small bead type particles. Interestingly, the surface of the polyacrylamide**–**silica composite material (Fig. 4f) is similar to the surface of polyacrylamide–alumina composite material (Fig. 4d), probably because of a similar type of synthesis mechanism. So from both images, it can be understood that the particle sizes increased by granulation as well as filling mechanism in the case of alumina and silica as filler particles, also supporting the FBRM results.

SEM images of neat zeolite Y (Fig. 4g) showed tiny particles on the surface whereas its corresponding polyacrylamide–zeolite Y composite material (Fig. 4h) showed larger particles on the surface. This result clearly reveals the formation of composites by granulation/agglomeration process of zeolite Y with *in situ* generated polyacrylamide moiety. Though the SEM results show a clear idea about the successful formation of composite materials, precipitation of formed products during the process may be the reason for controversial observation by FBRM analysis. Titania (Fig. 4i) showed tiny particles on surface whereas polyacrylamide–titania composite material (Fig. 4j) showed agglomerated tiny particles to larger particles as one kind of morphology along with crystal type morphology. These results showed that after larger particles formation, further process leaded to a well-ordered crystal type material, may be due to a precipitation process. So, the formation of well-ordered crystals during the process could be the reason for the drop in the particle size observed after certain time by FBRM analysis. SEM image of activated carbon (Fig. 4k) showed surfaces containing pores along with open type tunnel structures. In the case of polyacrylamide–activated carbon composites (Fig. 4l), it is clearly shown that the pores were filled by some foreign materials that may ended in the breakdown of tunnel structures into rigid structures. This result matches well with FBRM analyses, where a constant particle size in the lower particle size region was observed. Residual biomass (Fig. 4m) and the polyacrylamide–residual biomass composites (Fig. 4n) showed a flake like and broken flake like morphologies respectively. Both SEM images show that the surface as well as particle size of the formed composite materials are completely different from neat filler material. This change could be related to the adherence of the polymer matrix on the surface of the residual biomass, resulting in the continuous growth of the particle size in the composite materials as observed by FBRM analysis. SEM analysis of all the prepared polymer–filler composite materials clearly evidenced the formation of composite materials via *in situ* polymerization as supported by FBRM analysis too.

### Fourier-transform infrared spectra studies

Fourier-transform infrared (FT-IR) spectra of neat filler particles and the prepared polymer–filler composite materials are shown in Electronic Supplementary Information Fig. 1S. Neat montmorillonite showed small broad band around 1640 cm^−1^ corresponding to –OH bending vibration from water. Montmorillonite, alumina and their corresponding polyacrylamide composite materials showed a band in the range of 3300–3500 cm^−1^ indicating the presence of –OH groups. Montmorillonite and polyacrylamide–montmorillonite composite material showed characteristic bands around 1025 and 795 cm^−1^ that correspond to Si–O–Si stretching vibrations in both samples^34^. Silica and polyacrylamide–silica composite material also obeyed the characteristic Si–O–Si stretching vibrations as like montmorillonite and its corresponding polymer composite material. Zeolite Y and polyacrylamide–zeolite Y composite materials exhibited bands at ~1050 and ~790 cm^−1^ corresponding to asymmetric and symmetric stretching vibrations of external linkages^35^. Titania as well as its corresponding polyacrylamide composite material showed a band at around 3500 cm^−1^ corresponding to the stretching vibrations of –OH groups linked with titanium atoms (Ti–OH). Also the band around 1650–1500 cm^−1^ may be due to the bending vibrations of –OH groups from free or absorbed water molecules^36^. Activated carbon and its corresponding polyacrylamide composite materials showed a characteristic band around 1600 cm^−1^ supporting the presence of C=C stretching vibrations of aromatic rings^37^. Residual biomass and its corresponding polyacrylamide–composite material exhibited broad bands between 3600–3000 cm^−1^ corresponding to the hydrogen bonds of lignin and proteins moieties. Also, both samples exhibited bands in the region of 1200–1100 cm^−1^ corresponding to C–O–C stretching vibrations coming from the source components. The characteristic sharp bands around 1600 cm^−1^ support the presence of C=O stretching vibrations of protein moieties. Disappearance of bands between 860–930 cm^−1^ in the polyacrylamide–residual biomass composite materials indicate that –C–H groups (out-of-plane deformation) were affected by *in situ *polymerization process. FT-IR analysis of the product samples show bands around 3300–3200 cm^−1^ corresponding to –N–H stretching vibrations of polyacrylamide moiety. Also in some cases, the bands between 3500–3200 cm^−1^ represent the presence of –OH groups in the product composite materials same like in source filler particles. The bands in the region of 1690–1650 cm^−1^ indicate the presence of C=O amide groups of polyacrylamide moiety.

Powder X-ray diffraction (PXRD) results did not show any support for the formation of polyacrylamide–alumina composite materials (see Electronic Supplementary Information & Fig. 2S), most probably because of the small amount of polyacrylamide moiety and/or because of its amorphous nature.

### Particle size analysis

Independent particle size analysis of the filler particles and the prepared polyacrylamide–filler composite materials are given in Electronic Supplementary Information Table 1S. In all the cases, it is clearly observed that compared to the neat filler particles, the *in situ* formed polyacrylamide–filler composite materials resulted in larger particles of all diameters. These results support well the previous observations of FBRM technique. Interestingly, the independent particle size analysis of polyacrylamide–titania composites showed a huge increase compared to neat titania particles indicating that the increase in particle size by *in situ* polymerization process as evidenced by SEM analysis. However, because of the lack of dispersion or precipitation/solidification of the composite materials may found the difficulty in the FBRM analysis and that could be the reason for the observation of drop in particle size. Independent particle size analysis of activated carbon and polyacrylamide–activated carbon composites showed almost constant values (the changes are in the negligible value) and the trend is similar to FBRM analysis. Though the FBRM and independent particle size analysis results of residual biomass and its polyacrylamide composites are contradictory, dispersing the composite material in water medium (for independent particle size analysis) may breakdown some of the particles because of the loss of some stable organic moieties occured during the *in situ* polymerization process.

### Surface area & pore volume analyses

Surface characterization of some of the filler particles and their corresponding polymer–filler composite materials are given in Table 2. Montmorillonite showed surface area of 245 m²/g whereas polyacrylamide–montmorillonite composite showed a surface area of 112 m²/g. This result shows that *in situ* polymerization resulted in the formation of composite material by following the filling/coating process, leading to a drop in the surface area. Alumina as well as polyacrylamide–alumina composite showed a surface area of 236 and 84 m²/g respectively. Similarly, residual biomass and polyacrylamide–residual biomass composite showed a surface area of 0.8 and 0.1 m²/g respectively. In both cases, the surface area analyses follow the same trend as observed in *in situ* polymerization process of montmorillonite under the studied conditions. In all the cases neat filler materials showed high surface area compared to polyacrylamide–filler composite materials which indicates the successful granulation by the polymer moiety on filler particles during *in situ* polymerization. Also, these results match well with the previous observations on FBRM and SEM analyses for the same materials formation process. Furthermore, polyacrylamide–montmorillonite composite showed a pore volume of 0.148 cm^3^/g which is lower than neat montmorillonite (0.318 cm^3^/g). Similarly, compared to neat alumina (0.299 cm^3^/g) the resulted polyacrylamide–alumina composite showed a lower pore volume of 0.110 cm^3^/g. These results show that *in situ* polymerization process resulted in a drop in pore volume for the polyacrylamide–filler composites than individual filler particles. This observation supports the FBRM, SEM and surface area results on following the filling or granulation mechanism during the composites formation by *in situ* polymerization process. In the case of residual biomass, the observation of an increase in pore volume from 0.004 cm^3^/g to 0.005 cm^3^/g may be due the removal/escape of some organic moieties which could change the surface of the formed composites.

**Table 2:** Surface characterizations of some of the neat filler particles and the prepared polyacrylamide–filler composites.

Name of the Material	Surface Area (m²/g)	Pore volume (cm^3^/g)
Montmorillonite	245	0.318
Polyacrylamide–montmorillonite composite	112	0.148
Alumina	236	0.299
Polyacrylamide–alumina composite	84	0.110
Residual biomass	0.8	0.004
Polyacrylamide–residual biomass composite	0.1	0.005

### Thermogravimetric analysis

**Figure 5:**
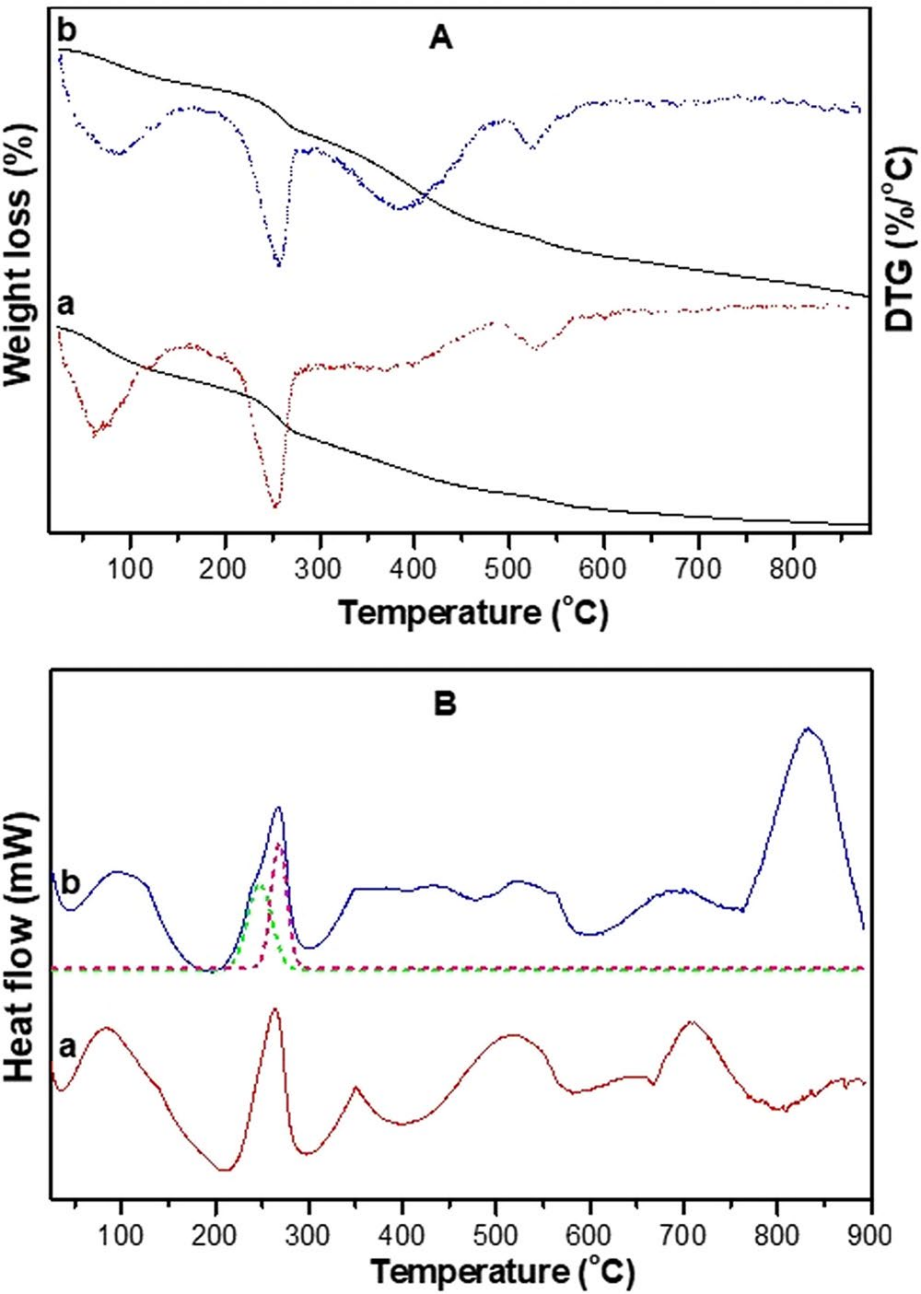
(**A**) TGA-DTG profiles of (a) alumina, (b) polyacrylamide–alumina composite. (**B**) DSC analysis of (a) alumina, (b) polyacrylamide–alumina composite; Dotted lines (–) indicate deconvoluted peaks.

Thermogravimetric analysis (TGA) profiles and first-derivative curves (differential thermogravimetric analyses, DTG) of alumina and polyacrylamide–alumina composite material are compared in Fig. 5A. The detailed weight loss corresponding to the different temperature ranges are given in Electronic Supplementary Information Table 2S. Both samples exhibited a small weight loss at temperatures of less than 200 °C that may correspond to physisorbed water molecules. In this temperature region, the weight loss of alumina (11%) is higher than the composite material (7%), indicating that the later may present water molecules trapped in the composite matrix. Though the second weight loss (200–300 °C) patterns are same in alumina and polyacrylamide–alumina composite materials, the later showed a slight drop (8%) in weight loss compared to alumina (10%). Also the polyacrylamide–alumina composite material showed a slight shift in peak maxima to 258 °C which is lower than the peak maxima of alumina. This second weight loss is probably due to the dehydration process in which polyacrylamide–alumina composite material ended in a less weight loss because of intra-molecular hydrogen bonding. The third weight loss in the temperature region of 300–500 °C, indicated that the polyacrylamide composite material (16%) resulted in a higher weight loss than neat alumina material (10%). In general, this temperature region corresponds to the weight loss of polyacrylamide moieties^38^ by releasing ammonia due to imidization reaction between the amide groups of monomer units^39^ and thus resulting in a higher weight loss of polyacrylamide–alumina composite materials. Additionally, in this region the structural transformation of boehmite (see PXRD results in Electronic Supplementary Information) to alumina by dehydration process is also possible. At temperatures of more than 500 °C, polyacrylamide–alumina composite materials resulted in higher weight loss (12%) than neat alumina material (5%). This result clearly indicates that higher weight loss for polyacrylamide–alumina composite material may be due to the complete decomposition of polymer matrixes along with some structural transformation of alumina matrixes.

Overall, alumina resulted in ~36% of weight loss after the analysis whereas the same in polyacrylamide–alumina composite materials was ~43%. This observation clearly indicates that presence of the additional polymer matrix in the formed polyacrylamide–alumina composite material resulted in a higher weight loss than neat alumina sample, also supporting the surface area analysis.

### Differential scanning calorimetric analysis

Differential scanning calorimetric (DSC) studies were performed to find out the crystallization properties of alumina and polyacrylamide–alumina composite materials and the results are shown in Fig. 5B. Pure alumina and polyacrylamide–alumina composite materials exhibited six endothermic peaks at the temperature ranges from 50–900 °C and the quantitative details are given in Table 3. Alumina showed a first broad endothermic peak maxima at 83.4 °C with a ∆H value of 292.1 J/g whereas polyacrylamide–alumina composite material showed the same at 93.7 °C with a ∆H value of 200.1 J/g. The drop in the ∆H value corresponds to the loss of less amount of water from polyacrylamide–alumina composite material as evidenced by the TGA analysis. The ∆H value for the second peak in alumina and polyacrylamide–alumina composite materials were 220.1 and 245.7 J/g respectively, which also corresponds to the dehydration process. The small shoulder peak at 246.6 °C in polyacrylamide–alumina composite material is probably related to the removal of different type of water molecules from the whole matrix that may have been increased the ∆H value. The third endothermic peak around 350 °C in alumina may be due to the melting point temperature (Tm) of the AlO(OH) phase (see PXRD results in Electronic Supplementary Information) present in it. The same in polyacrylamide–alumina composite material shifted to a higher temperature of 402 °C may be because of the change in polyacrylamide melting point temperature due to structural transformations. Also, probably the polyacrylamide–alumina composite material undergone the removal of NH_3_ by imidization reaction from the polyacrylamide that may have been increased the ∆H value compared to alumina in the same region. Though both materials showed an endothermic peak around 518 °C, the ∆H value for alumina and polyacrylamide–alumina composite materials were 266 and 101.9 J/g respectively. The endothermic peak around 518 °C in alumina is related to a sequence of various structural transformations, resulting in a drop of the ∆H value for polyacrylamide–alumina composite materials. In polyacrylamide–alumina composite materials, the higher temperature of the endothermic peaks may be due to the breakdown of polyacrylamide backbones and formation of nitriles and long-chain hydrocarbons^39^ that could be related to higher ∆H values such as 104.2 and 595.3 J/g. In all these temperature regions, along with polymer denaturation, basic alumina structural transformations are also possible as observed in neat alumina. All these results support that the presence of polyacrylamide moiety could be the reason for the increase in the thermal stability of the polyacrylamide–alumina composite material compared to alumina.

**Table 3:** Quantitative DSC measurements of alumina and polyacrylamide–alumina composites.

Name of the material	First peak		Second peak		Third peak		Fourth peak		Fifth peak		Sixth peak	
	Peak maxima (°C)	∆H (J/g)	Peak maxima (°C)	∆H (J/g)	Peak maxima (°C)	∆H (J/g)	Peak maxima (°C)	∆H (J/g)	Peak maxima (°C)	∆H (J/g)	Peak maxima (°C)	∆H (J/g)
Alumina	83.4	292.1	263.4	220.1	350.7	81.6	518.2	266.0	645.0	14.5	706.4	157.6
Polyacrylamide–alumina composite	93.7	200.0	(246.6 & 267.5)^a^	245.7^b^	402.4	167.5	517.9	101.9	694.5	104.2	831.2	595.3

^a^Peak maxima for two different peaks, ^b^Addition of the ∆H values of two peaks.

## Conclusions

Real-time particle size and particle size distributions were analysed by focused beam reflectance measurement (FBRM) during the preparation of various composites consisting in filler particles and polyacrylamide moieties at 80 °C for 24 h via *in situ* polymerization process. FBRM results followed different trends depending upon the nature of filler particles as well as the formed composites and proved that particle size kinetic changes were unique for each process. Out of the filler particles studied, montmorillonite, alumina and residual biomass resulted in a continuous increase in the particle size at <1000 μm scale with respect to the time. Interestingly, other filler particles such as silica, zeolite Y, titania and activated carbon resulted in random observations in which the particle sizes remain unchanged after reaching the optimum time of the process. In all cases, SEM results clearly revealed that the surface of the formed composites were completely different from neat filler particles, supporting FBRM observations. Additionally, SEM analyses clearly evidenced that various mechanisms such as granulation, coating and filling were involved in the preparation of the composites during *in situ* polymerization process. Elemental (CHNS-O), FT-IR and particle size analyses also gave further support to the formation of polyacrylamide–filler composites under the studied conditions. The drop in the surface area observed for montmorillonite and alumina based polyacrylamide composites revealed that *in situ* polymerization approach followed a multiple step formation process due to the presence of thermoset plastic matrix in the system towards the fabrication of polyacrylamide–filler composites. Pore volume of the prepared montmorillonite and alumina based polyacrylamide composites were lower than neat filler particles, supporting structural changes in the formed composites. Thermal analyses of alumina and polyacrylamide–alumina composites proved that the thermal stability of the later sample increased compared to neat filler particles, further supporting that the nature of both samples were completely different.

## Experimental Section

### Materials

Acrylamide (98.0%), N,N′-methylene bisacrylamide (N,N′-MBA), ammonium persulfate (APS), montmorillonite and titanium (IV) oxide (Titania; TiO_2_) were purchased from Sigma-Aldrich. Alumina was prepared from aluminum isopropoxide (Sigma-Aldrich) in the laboratory. Zeolite Y (Si/Al = 80) and activated carbon were purchased from Zeolyst International, Netherlands and Alkaloid, Skopje respectively. Lessonia Trabeculata (macroalgae) biomass was collected from coastal region of Chile and from that polymers and pigments were removed for specific applications. The remaining residual biomass was used for this study. Residual macroalgal biomass composition is given in Electronic Supplementary Information Table 3S.

### Polyacrylamide–filler composite materials preparation process

The polyacrylamide–filler composite materials fabrication process was carried out on a Mettler-Toledo RC1 system. In a reactor vessel, 100 mL of water was taken and then FBRM probe was immersed into the system. After that the reactor vessel was heated up to 80 °C by passing hot silicone oil in the double jacketed setup. The content in the flask was mixed well by using an overhead stirrer with the stirring speed of 400 rpm. 4 g of filler particle sources, 2 g of acrylamide and 1 g of N,N′-MBA (cross linker) were mixed well and added to the system after reaching the desired temperature. After 5 minutes, 150 mg of APS (initiator) was added to initiate the polymerization process. The reaction was performed for 24 h and the on-line FBRM data were measured during the whole process. Finally, the formed processed sample was collected and washed with excess water followed by oven drying at 70 °C for overnight.

### On-line particle size analysis

On-line particle size was measured by placing the FBRM probe in the RC1 reactor system and in all the studies, the probe was cleaned and stabilized well with water for zero particle counts. All the FBRM measurements were performed over 10 second periods for the number of counts ranging between 15 and 1000 μm size. On-line particle size distribution was measured by changing the options in the software.

### Characterization techniques

The CHNS-O analysis was carried out using EURO EA Elemental Analyzer. The sample weighed in milligrams housed in a tin capsule was dropped into a quartz tube at 1020 °C with constant helium flow (carrier gas). Oxygen was calculated by difference with 100%. Scanning electron microscope of the samples were performed using electronic microscope FE–SEM SUPRA 35–F (Carl Zeiss) with energy-dispersive spectrometer Inca 400 (Oxford Instruments). All the analyses were carried out with an accelerating voltage of 1 Kv and a working distance of 4–5 mm. Fourier-transform infrared spectra were recorded on FTIR Spectrum 100 (Perkin Elmer) with the Universal ATR (UATR) Accessory (diamond cell) using 4 scans with the resolution of 4 cm^−1^ which were accumulated and averaged to improve the signal-to-noise ratio. Thermogravimetric analysis and differential scanning calorimetry were performed simultaneously (STA 6000 Perkin Elmer) under nitrogen atmosphere (50 mL/min) from 25 to 900 °C using a heating rate of 20 °C/min. Particle size analysis of the materials was performed on Shimadzu Sald-3101, laser diffraction particle size analyser by dispersing the samples in water medium. The analysis was done three times consecutively and the average values are reported. Surface area and pore volume of the samples were assessed by nitrogen adsorption on NOVA 1000e surface area analyser and the data were processed by using the published methods.

## Supplementary information

Real-time Particle Size Analysis Using the Focused Beam Reflectance Measurement Probe for In Situ Fabrication of Polyacrylamide–Filler Composite Materials
