# Supplementary material for: Real-time Particle Size Analysis Using the Focused Beam Reflectance Measurement Probe for In Situ Fabrication of Polyacrylamide–Filler Composite Materials
Source: Sci Rep. 2019 Jul 12;9:10126. doi: 10.1038/s41598-019-46451-x (PMC6625984; doi:10.1038/s41598-019-46451-x)
Supplement: Supplementary file 1 — Real-time Particle Size Analysis Using the Focused Beam Reflectance Measurement Probe for In Situ Fabrication of Polyacrylamide–Filler Composite Materials [file 41598_2019_46451_MOESM1_ESM.pdf]

Electronic Supplementary Information

Real-time Particle Size Analysis Using the Focused Beam Reflectance Measurement Probe for *In Situ* Fabrication of Polyacrylamide–Filler Composite Materials

Sivashunmugam Sankaranarayanan[a,b,]*, Blaž Likozar[b], Rodrigo Navia[a,c,d]

[a]Scientific and Technological Bioresource Nucleus (BIOREN), Universidad de La Frontera, Av. Francisco Salazar 01145, Temuco, Chile.
[b]Department of Catalysis and Chemical Reaction Engineering, National Institute of Chemistry, Ljubljana, Slovenia.
[c]Department of Chemical Engineering, Faculty of Engineering and Sciences, [d]Centre for Biotechnology and Bioengineering (CeBiB), Universidad de La Frontera, Av. Francisco Salazar 01145, Temuco, Chile.

Correspondence:

Sivashunmugam Sankaranarayanan, Scientific and Technological Bioresource Nucleus, Universidad de La Frontera, Temuco, Chile

E-mails:

Sivashunmugam Sankaranarayanan: sivashunmugams@gmail.com; sivas.sanka@ufrontera.cl; Blaž Likozar: blaz.likozar@ki.si; Rodrigo Navia: rodrigo.navia@ufrontera.cl

Table 1S. Particle size analysis of the neat filler particles and the prepared polyacrylamide–filler composites

**Material**	**Cumulative diameter distribution (μm)**		
	**25 %**	**50 %**	**75 %**
Montmorillonite	0.4	0.5	0.6
Polyacrylamide–montmorillonite composite	3.5	10.4	28.9
Alumina	0.4	0.4	0.5
Polyacrylamide–alumina composite	1.0	3.1	42.7
Silica	4.4	8.4	15.8
Polyacrylamide–silica composite	6.0	20.1	68.7
Zeolite Y	0.5	1.1	2.4
Polyacrylamide–zeolite Y composite	1.1	2.7	6.4
Titania	0.6	0.6	0.7
Polyacrylamide–titania composite	324.9	444.2	545.2
Activated carbon	6.6	13.9	28.4
Polyacrylamide–activated carbon composite	6.6	13.0	24.8
Residual biomass	190.5	299.3	388.8
Polyacrylamide–residual biomass composite	186.2	282.6	369.5

Table 2S. Quantitative weight loss of alumina and polyacrylamide–alumina composites

Name of the material	Weight loss (%)				
	< 200°C	200-300°C	300-500°C	> 500°C	Total
Alumina	11	10	10	5	36
Polyacrylamide–alumina composite	7	8	16	12	43

Table 3S. Composition of residual macroalgal biomass

Test	Dry Basis (%)	As received (%)
Dry Matter	-	86.53
Moisture	-	13.47
Protein (N x 6.25)	10.81	9.35
Acid Detergent Fibre	24.20	20.94
Neutral Detergent Fibre	46.36	40.12
Lignin	6.58	5.69

FT-IR studies:

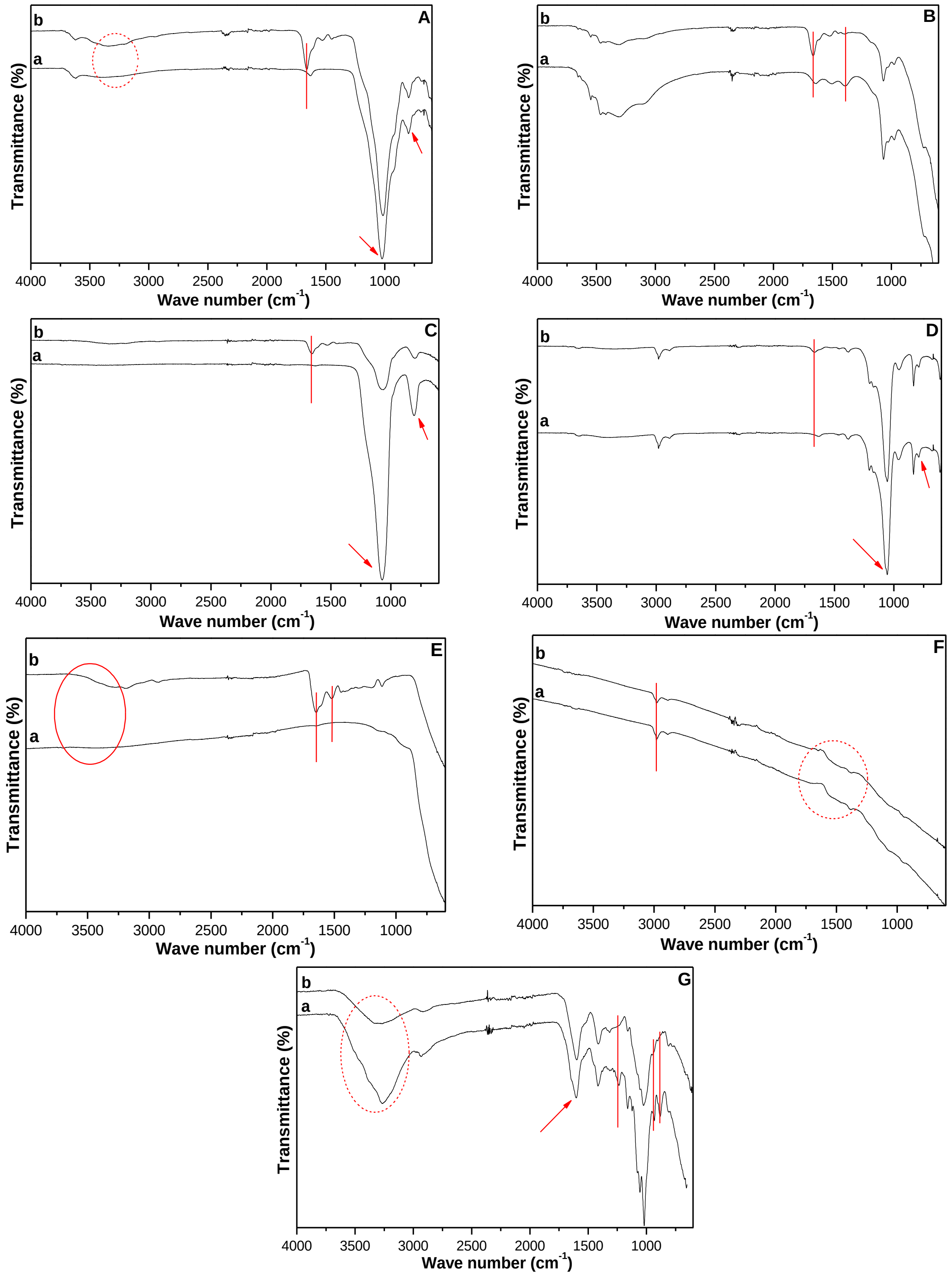

Fig. 1S. FT-IR analysis of (A) montmorillonite, (B) alumina, (C) silica, (D) zeolite Y, (E) titania, (F) activated carbon, (G) residual biomass; (a) Neat filler particles, (b) polyacrylamide–filler composites.

Powder X-ray diffraction analysis:

Powder X-ray diffraction was carried on a X-ray powder diffractometer PANalytical X'Pert PRO MPD with Cu Kα (1.5406 Å) radiation. The operating voltage and current were 45 kV and 40 mA respectively. The PXRD patterns were recorded at room temperature at a step size of 0.034° over a 2θ range of 2-80°. Alumina as well as polymer-alumina composite materials showed peaks at 2 theta values of 13.6, 27.8, 37.9, 49.0, 64.6 and 71.7° corresponding to boehmite (AlO(OH))[1] phase (*JCPDS No. 01-083-2384*). This result is in concordance with the FT-IR observations, showing the presence of -OH groups (~3450-3500 cm^{-1}) in alumina and polyacrylamide-alumina composite materials. Additionally, the peak at 53.0° indicated the presence of α-alumina phase (*JCPDS No. 46-1212*) in both materials. The diffraction peaks in alumina at 13.4 and 27.7° are slightly shifted to higher 2 theta values of 13.8 and 27.9° in the alumina-polymer composite. Due to its amorphous nature, polyacrylamide phase did not show any characteristic peak in PXRD analysis.

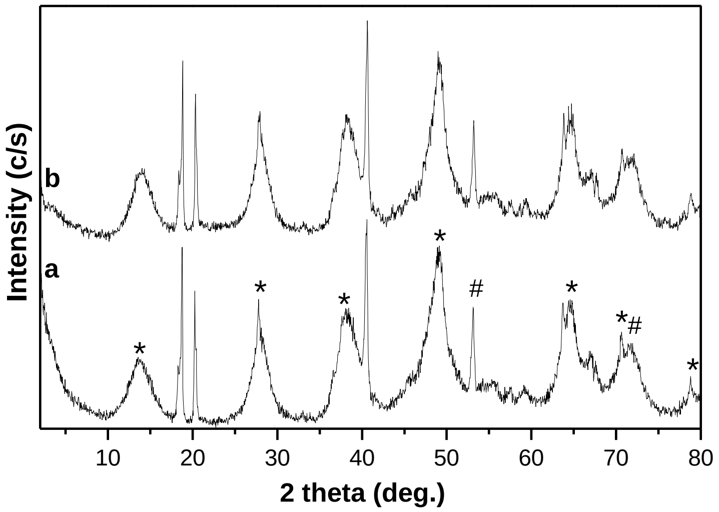

Fig. 2S. PXRD of (a) alumina and (b) polyacrylamide-alumina composite material. (*): boehmite (AlO(OH)) phase, (#): α-alumina phase.

1. Santos P. d. S., Coelho A. C. V., Santos H. d. S. & Kiyohara P. K. Hydrothermal synthesis of well-crystallised boehmite crystals of various shapes. *Mater. Res.* 12 (4), 437-445 (2009).
